# P-886. Impact of Pre-Checked Empiric Antibiotics for Suspected Sepsis in the Emergency Department

**DOI:** 10.1093/ofid/ofaf695.1094

**Published:** 2026-01-11

**Authors:** Lauren M Fasth, Micah A Jacobs, Adam Greenfield, Megan A Baumgartner, Frank D’Amico

**Affiliations:** University of Pittsburgh Medical Center, Pittsburgh, Pennsylvania; Romano, Panther and Assoc, PITTSBURGH, Pennsylvania; UPMC St. Margaret, Pittsburgh, Pennsylvania; UPMC St. Margaret, Pittsburgh, Pennsylvania; University of Pittsburgh Medical Center, Pittsburgh, Pennsylvania

## Abstract

**Background:**

1.7 million adults develop sepsis annually leading to more than 350,000 deaths in the United States. Although empiric cefepime is appropriate for some high-risk patients, it may provide unnecessary broad-spectrum coverage and contribute to increased antimicrobial resistance for patients without risk factors for multidrug-resistant organisms (MDROs). The purpose of this study was to demonstrate how empiric antibiotic selection changed at a community hospital emergency department (ED) before and after cefepime became the pre-selected antibiotic in the ED sepsis order set.

**Figure:**
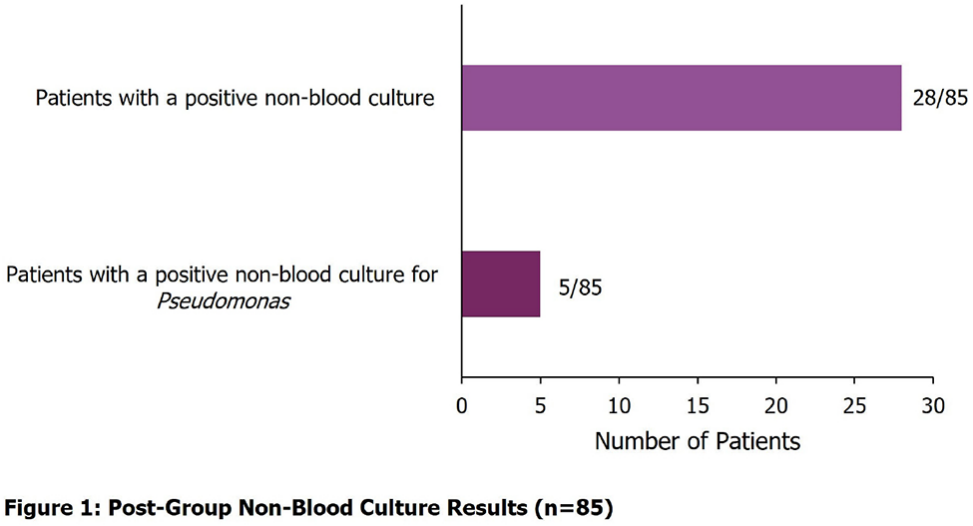

**Figure:**
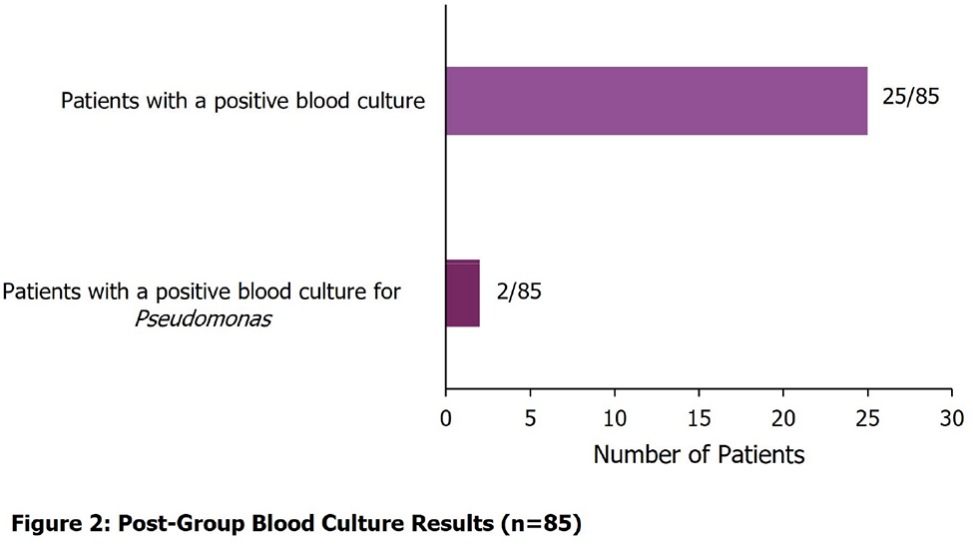

**Methods:**

This retrospective chart review included adults admitted to a community hospital, initiated on an empiric antibiotic, and ordered the ED sepsis order set. Data was collected pre- and post-September 2023 following order set modification to include pre-selected cefepime. Prior to September 2023, the order set did not include a pre-selected antibiotic. Chi-square test was used to determine the difference in cefepime administration rates. The primary outcome was frequency of cefepime administration before and after order set modification. The secondary outcome evaluated the appropriateness of empiric cefepime, as determined by patient risk factors for MDROs and culture results.

**Figure:**
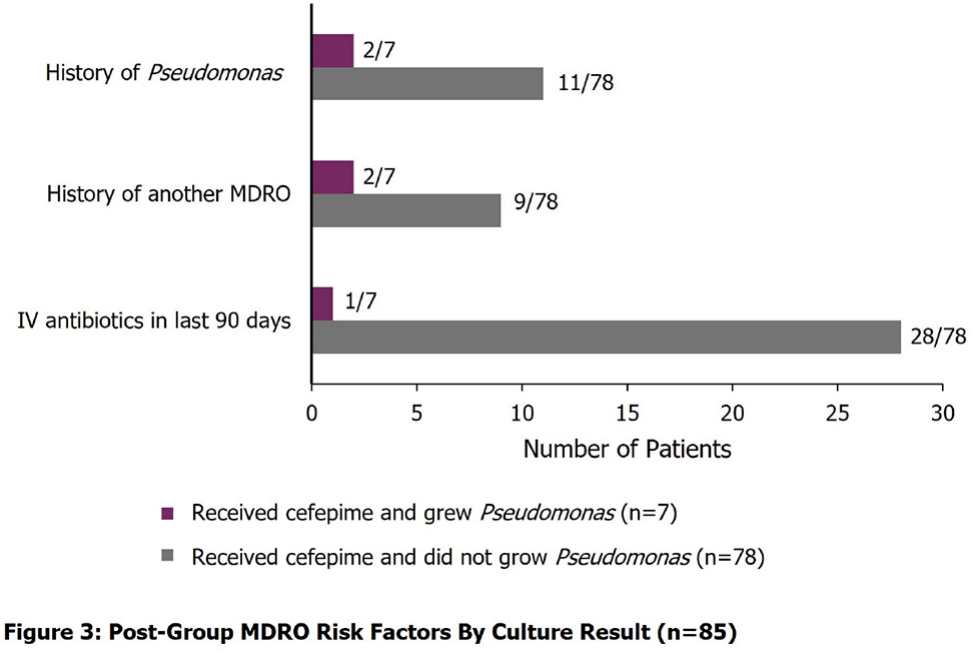

**Figure:**
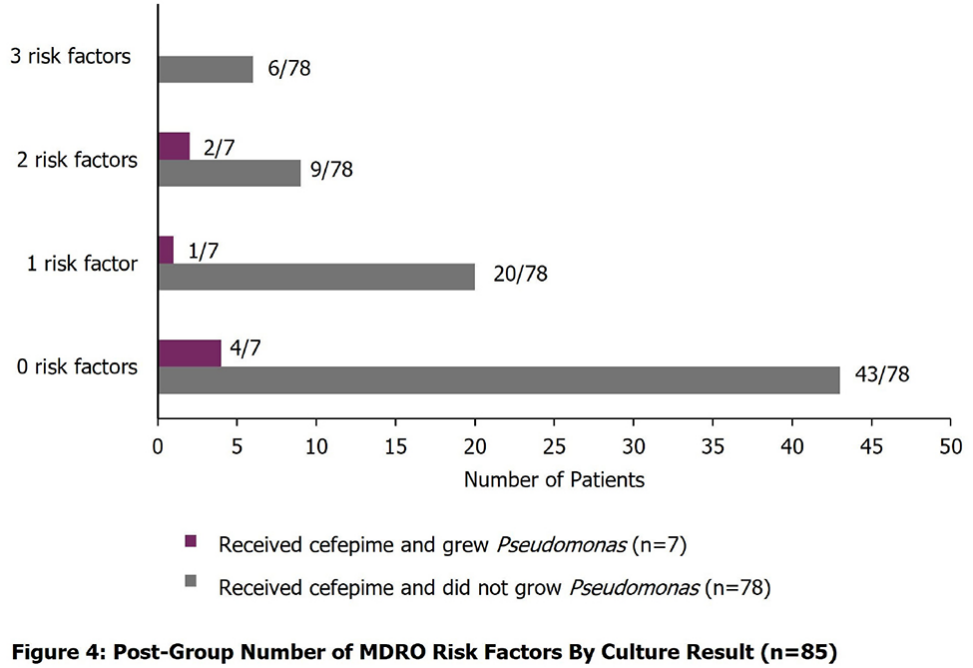

**Results:**

457 patients met inclusion criteria for the study (n=200 in the pre-group and n=257 in the post-group). Severity of illness and demographics were similar between the two groups. 16 patients (8%) received empiric cefepime in the pre-group and 85 (33.07%) received empiric cefepime in the post-group (p< 0.05), resulting in a 300% increase in empiric cefepime for suspected sepsis with the updated order set, 95% CI for the difference in rates, [0.18-0.33]. Among patients in the post-group who received cefepime, 8% of patients (n=7) grew *Pseudomonas* in a blood or non-blood culture (Figures 1 and 2). Risk factors for MDROs by culture result are described in Figures 3 and 4.

**Conclusion:**

Low rates of *Pseudomonas* on culture do not support the need for empiric cefepime for most patients in a community hospital setting. Data from this study may be used to support improved antimicrobial stewardship with empiric antibiotic selection driven by local antibiogram data and individual patient risk factors for MDROs.

**Disclosures:**

Micah A. Jacobs, MD, Abbvie: Honoraria|Cumberland pharmaceuticals: Honoraria|Merck: Grant/Research Support

